# The joint effect of congenital hypothyroidism and hypercaloric diet consumption as triggers of type 2 diabetes mellitus

**DOI:** 10.1530/ETJ-21-0050

**Published:** 2021-11-18

**Authors:** Jorge Alberto Tapia-Martínez, Margarita Franco-Colín, Vanessa Blas-Valdivia, Edgar Cano-Europa

**Affiliations:** 1 Laboratorio de Metabolismo I, Departamento de Fisiología, Escuela Nacional de Ciencias Biológicas, Instituto Politécnico Nacional, Colonia Unidad Profesional Adolfo López Mateos, Delegación Gustavo A. Madero, Ciudad de México, México; 2 Laboratorio 6, Departamento de Farmacobiología, Centro de Investigación y de Estudios Avanzados-Instituto Politécnico Nacional, Delegación Tlalpan, Ciudad de México, México; 3 Laboratorio de Neurobiología, Departamento de Fisiología, Escuela Nacional de Ciencias Biológicas, Instituto Politécnico Nacional, Colonia Unidad Profesional Adolfo López Mateos, Delegación Gustavo A. Madero, Ciudad de México, México

**Keywords:** congenital hypothyroidism, diabetes, metabolic syndrome, hypercaloric diet

## Abstract

**Introduction:**

Congenital hypothyroidism affects metabolic and thyroid programming, having a deleterious effect on bodyweight regulation promoting metabolic diseases. This work aimed to demonstrate the development of type 2 diabetes mellitus (T2D) in animals with congenital hypothyroidism, only by the consumption of a mild hypercaloric diet in the extrauterine stage.

**Methods:**

Two groups of female Wistar rats (*n  = *9): euthyroid and hypothyroid were used. Hypothyroidism was induced by a thyroidectomy with parathyroid reimplantation. Male offsprings post-weaning were divided into four groups (*n  = *10): euthyroid, hypothyroid, euthyroid + hypercaloric diet, and hypothyroid + hypercaloric diet. The hypercaloric diet consisted of ground commercial feed plus 20% lard and was administered until postnatal week 40. Bodyweight and energy intake were monitored weekly. Also, metabolic and hormonal markers related to cardiovascular risk, insulin resistance, and glucose tolerance were analyzed at week 40. Then, animals were sacrificed to perform the morphometric analysis of the pancreas and adipose tissue.

**Results:**

T2D was developed in animals fed a hypercaloric diet denoted by the presence of central obesity, hyperphagia, hyperglycemia, dyslipidemia, glucose tolerance, insulin resistance and hypertension, as well as changes in the cytoarchitecture of the pancreas and adipose tissue related to T2D. The results show that congenital hypothyroid animals had an increase in metabolic markers and an elevated cardiovascular risk.

**Conclusions:**

Congenital hypothyroid animals develop T2D, having the highest metabolic disturbances and a worsened clinical prognosis than euthyroid animals.

## Introduction

Thyroid hormones participate in several processes related to growth, development, cell differentiation, and metabolism in all cells from embryonic development to adulthood (1). The thyroid hormone deficiency during the prenatal stage causes congenital hypothyroidism that alters thyroid and metabolic programming. This idea is supported by the fetal origin of the metabolic diseases hypothesis (2, 3). Thus, metabolic diseases have their origin during the early life stages when the fetal physiological systems program the metabolism (4), and the extrauterine conditions are responsible for modulating the expression of the epigenetic changes (5, 6).

Some authors speculate that congenital hypothyroidism has an association with type 2 diabetes mellitus (T2D) development (7), but until now neither in rats nor in humans, this hypothesis has been proved. In humans, some meta-analyses reveal an association between hypothyroidism, obesity, and dyslipidemia; however, it is necessary for more studies because multifactorial situations distort the association between the variables (8, 9, 10, 11). On the other hand, previously in animal models, we reported the increased risk to develop T2D in congenital hypothyroid rats because they develop metabolic syndrome even when they are fed with a standard-balanced diet (12, 13, 14). The mentioned studies reveal that congenital hypothyroidism modifies the metabolic programming promoting dyslipidemia and hyperleptinemia with a change in the thyroid gland’s function (12, 15). These conditions could be seen severely increased when a hypercaloric diet is administered because it induces epigenetic methylation patterns in adipocyte-associated metabolic dysfunction (16).

For all the above-mentioned, this work aims to demonstrate that congenital hypothyroidism modifies metabolic programming and promotes T2D development when the animals consume a mild obesogenic diet in the extrauterine stage also to determine if this perturbation worsens the clinical prognosis compared to the euthyroid group.

## Materials and methods

### Experimental design

Eighteen virgin Wistar female rats were conditioned in controlled conditions (12 h light:12 h darkness cycle, temperature: 21 ± 1 °C) and maintained with food and water *ad libitum.* After a week of conditioning, animals were randomly divided into two mothers groups (*n =* 9): (i) euthyroid and (ii) hypothyroid.

Hypothyroidism was induced surgically by a thyroidectomy with parathyroid reimplantation in the hypothyroid group as previously described (12, 13, 14, 17). Seven days post-surgery, three females were placed with one male for mating. One day after birth, eight pups were randomly assigned to their corresponding euthyroid or hypothyroid mother during lactation. Two weeks after weaning, male offsprings were divided into the following experimental groups (*n* = 10): (ii) euthyroid, (ii) hypothyroid, (iii) euthyroid + hypercaloric diet, and (iv) hypothyroid + hypercaloric diet. Then, they were placed into individual cages (20 × 30 × 18 cm), with access to water and food *ad libitum* for 40 weeks. The hypercaloric diet was prepared by mixing grounded commercial feed (LabDiet, 5001) with 20% lard. The composition of the diet is shown in Table 1.

**Table 1:** Composition of the purine diet and the hypercaloric diet added with 20% lard.

Component of the diet	Purine diet	Hypercaloric diet (%)
Proteins	24.1	19.28
Carbohydrates	57.94	46.35
Lipids	5	24
Crude fiber	5.2	4.16
Minerals	6.9	5.52
Sodium	0.39	0.31
Energy supply (kJ/g)	16.73	20.92

Bodyweight and energy intake were measured weekly until the end of the experiment. The systolic blood pressure was measured at week 40 in conscious rats by a non-invasive method with a digital plethysmograph coupled to the rat tail (Le 5002, Panlab–Harvard apparatus). The number of deaths was quantified throughout the experiment to obtain the survival percentage.

### Determination of glucose tolerance and insulin resistance

Tests were performed at the beginning of week 40 as 1 per day, after 6 h of fasting. For the insulin resistance test, each animal received 0.75 IU/kg of rapid-acting insulin intraperitoneally. Meanwhile, for the glucose tolerance test, they received 1.8 g/kg of dextrose intraperitonally. After that, blood glucose levels were monitored at 0, 30, 60, and 120 min using a glucometer (Abbott®).

### Metabolic and hormonal parameters

Two days after the glucose homeostasis test, animals were fasted for 6 h, and blood samples from the tail vein were obtained, centrifuged at 30,000 ***g*** for 20 min to obtain serum, which was individually kept at −20°C until assay. Metabolic parameters such as glucose, triglycerides, cholesterol, HDL-c (high-density lipoprotein), LDL-c (low-density lipoprotein), VLDL-c (very low-density lipoprotein) were measured using RANDOX® kits. Besides, the Castelli index I and II was obtained using the following calculations (18):


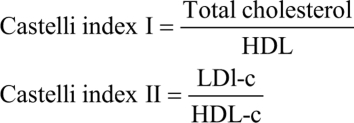


Hormonal profile was assessed using ELISA commercial kits for insulin (LINCO®), T_3_ (DIAGMEX®), T_4_ (DIAGMEX®), and leptin (Millipore®). Also, the insulin sensitivity index (ISI) (19) and homeostatic model assessment of insulin resistance (HOMA-IR) were calculated as previously described (20, 21):


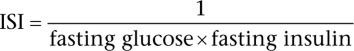



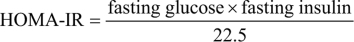


The values obtained in the determinations were compared with cut-off values for metabolic syndrome, insulin resistance, and T2DM as previously reported (22, 23).

### Histological analysis

At the end of week 40, the animals were sacrificed with monosodic pentobarbital (35 mg/kg intraperitoneally), and the adipose tissue was dissected and weighted to determine adiposity percentage. A portion of the pancreas and visceral adipose tissue was fixed in a 10% buffered formalin for 48 h and was embedded in paraffin. Five-micrometer section slices for the pancreas and 20 µm for visceral adipose tissue were obtained with a standard microtome (LEICA RM 2145). The slices were stained with hematoxylin–eosin, and the photomicrographs acquired with the Nikon-50i microscope were analyzed with ImageJ software that quantified the Langerhans islets per 1 cm^2^ and counted the cellularity of each one. Also, it quantified adipocytes per+ 1 cm^2^ and its diameter.

### Statistical analysis

All the variables except the adiposity levels and survival percentage are presented as the mean ± s.e.m., and they were evaluated by repeated-measure two-way ANOVA and Student-Newman-Keuls *post hoc* test. It considered the diet and thyroid state as factors. The adiposity levels represent the median ± interquartile spaces. The adiposity levels and area under the curve (AUC) were evaluated by the Kruskal–Wallis test. Finally, the survival percentage was measured by test Log-Rank (Mantel-Cox). *P* < 0.05 was considered statistically significant.

## Results

The composition of the standard and the hypercaloric diet added with 20% lard is shown in Table 1. It is noted that the hypercaloric diet provides a higher energy content, mostly given by lipid content.

**Figure 1:**
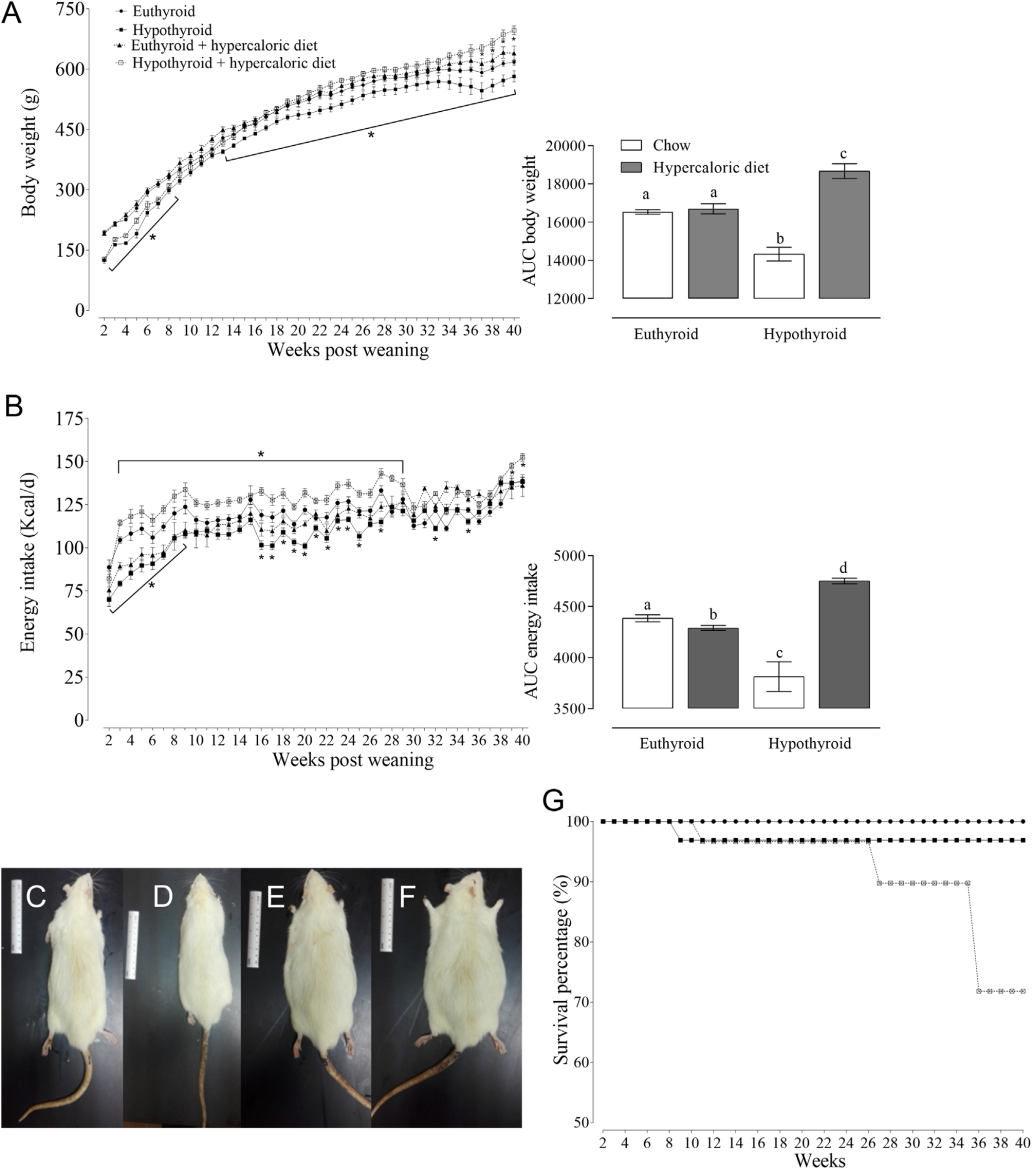
Bodyweight (A) and energy intake (B) during 42 weeks after weaning. The bar graphs represent the area under the curve for each of the treatments. Data represent the mean ± s.e.m. *P< 0.05 vs the euthyroid group. RM-two-way ANOVA and Student–Newman–Keuls *post hoc*. The AUC was evaluated by the Kruskal–Wallis test (*P <* 0.05*;* a ≠ b ≠ c) equal letters indicate that there is no difference between groups, and different letters indicate statistical difference. Graph G represents the survival percentage during the treatment, *P < 0.05log-rank (Mantel-Cox) test. The photographs are representative of the euthyroid (C), hypothyroid (D), euthyroid + hypercaloric diet (E), and hypothyroid + hypercaloric diet (F) at the end of week 40.

Figure 1 presents the results of the bodyweight (panel A), energy intake (panel B), as well as representative photographs of the animals at the end of the treatment (panels C–F). The hypothyroid animals fed with a chow standard diet presented lower bodyweight and energy intake compared with the other groups. Euthyroid animals fed a 40-week hypercaloric diet had a mild increase in their bodyweight with a mild reduction in their energy intake. Meanwhile, the congenital hypothyroid animals with the hypercaloric diet had a marked increase in their bodyweight since week 34 of treatment with an increase in their energy intake, presenting central obesity. Finally, panel G shows the animals' survival percentage during the experiment. The euthyroid animals fed chow and a hypercaloric diet had a 0% mortality rate. However, the hypothyroid group had a 2.94% mortality, and the hypothyroid fed with a hypercaloric diet group had 27.15% mortality. The necropsy analysis showed that most of the congenital hypothyroid animals that died presented fulminant acute myocardial infarction.

Table 2 shows that congenital hypothyroidism develops metabolic syndrome by the presence of hyperglycemia, hypertriglyceridemia, hypercholesterolemia, hyperleptinemia, hyperinsulinemia, and insulin resistance with an increase in cardiovascular risk without systolic pressure alteration, compared with euthyroid animals. Meanwhile, the hypercaloric diet causes hypertension and it enhances all these markers. These results were associated with T2D development. Also, the congenital hypothyroid animals fed a high-fat diet presented the highest levels in all metabolic and hormonal markers with the highest elevated cardiovascular risk. Congenital hypothyroidism caused a reduction in the levels of T_3_ and T_4_, while the hypercaloric diet did not modify the levels of thyroid hormones. Also, in previous studies, we have reported the status of thyroid function at various stages of development using the same experimental model as the one used for the present work. The results obtained show that thyroid function in congenital hypothyroid pups remains low throughout development (12, 13, 14).

**Table 2:** Metabolic, hormonal, insulin resistance, and cardiovascular risk markers.

	Euthyroid	Hypothyroid	Euthyroid + hypercaloric diet	Hypothyroid + hypercaloric diet
Glucose (mg/dL)	108.09 ± 4.37^a^	144.21 ± 3.26^b^	199.41 ± 2.04^b^	235.15 ± 3.63^c^
Triglycerides (mg/dL)	128.43 ± 6.08^a^	215.16 ± 13.36^b^	282.45 ± 10.09^b^	325. 01 ± 28.02^b^
Cholesterol (mg/dL)	135. 43 ± 2.40^a^	178.15 ± 7.64^b^	193.32 ± 4.12^b^	218.13 ± 8.17^c^
HDL-c (mg/dL)	32.09 ± 2.87^a^	42.96 ± 1.54^a^	41.60 ± 0.21^b^	51.37 ± 0.80^b^
LDL-c (mg/dL)	52.71 ± 1.09^a^	70.82 ± 3.21^b^	77.40 ± 0.71^c^	84.70 ± 1.59^d^
VLDL-c (mg/dL)	32.06 ± 4.12^a^	43.07 ± 3.13^a^	47.07 ± 1.64^b^	51.51 ± 2.34^b^
NEFA (mmol/L)	0.390 ± 0.01^a^	0.386 ± 0.04^b^	0.558 ± 0.15^b^	0.334 ± 0.04^a^
Castelli I index	1.95 ± 0.93^a^	5.20 ± 0.86^b^	7.18 ± 0.28c	11.87 ± 0.67^d^
Castelli II index	0.49 ± 0.06ª	2.73 ± 0.87^b^	4.87 ± 0.28^c^	7.76 ± 0.21^d^
T_3_ pups day 21 (ng/dL)	121.85 ± 0.11^a^	95.36 ± 0.09^b^	–	–
T_4_ pups day 21 (µg/dL)	10.32 ± 0.13^a^	8.12 ± 0.18^b^	–	–
T_3_ (ng/dL)	104.34 ± 5.37^a^	83.78 ± 3.89^b^	106.46 ± 1.68^a^	80.34 ± 2.87^b^
T_4_ (µg/dL)	9.20 ± 0.14^a^	7.50 ± 0.08^b^	9.06 ± 0.20^a^	7.36 ± 0.29^b^
Leptin (ng/dL)	2.27 ± 0.02^a^	3.17 ± 0.05^b^	4.28 ± 0.02^a^	5.56 ± 0.03^b^
Insulin (ng/dL)	0.84 ± 0.02^a^	2.07 ± 0.01^b^	3.89 ± 0.05^a^	4.13 ± 0.07^b^
HOMA-IR	1.67 ± 0.2^a^	3.08 ± 0.12^b^	4.77 ± 0.32^c^	6.06 ± 0.12^d^
ISI	0.0095 ± 0.0001ª	0.0028 ± 0.0002^b^	0.0013 ± 0.0001^c^	0.0011 ± 0.0004^d^
Systolic blood pressure (mmHg)	118.76 ± 5.43^a^	123.75 ± 8.45^a^	144.98 ± 7.95^b^	186.98 ± 5.56^c^

Data represent the mean ± s.e.m. Two-way ANOVA and Student–Newman–Keuls *post hoc*; a≠b≠c≠d, *P<* 0.05, equal letters indicate that there is no difference between groups, and different letters indicate the statistical difference.

**Figure 2:**
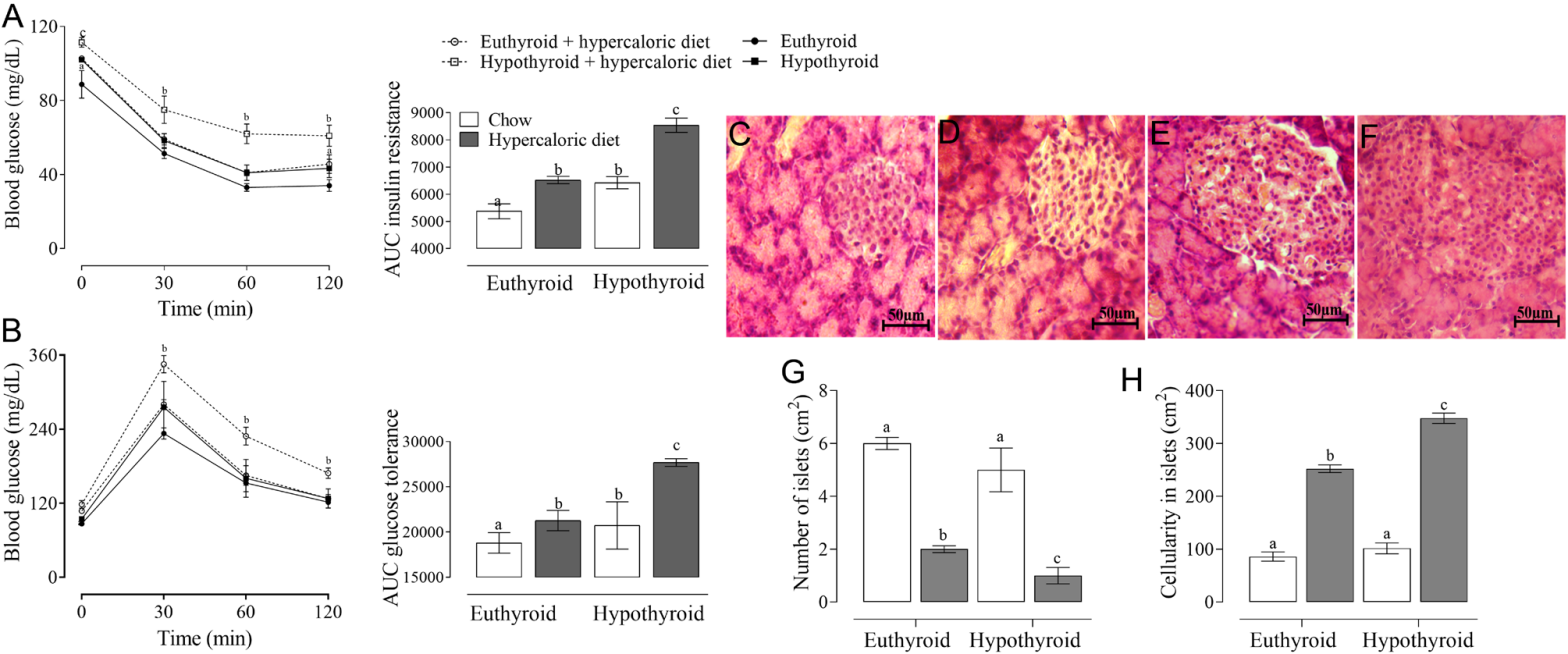
Insulin resistance (A) and glucose tolerance (B) at week 40, the bar graphs represent the AUC. Photomicrography of pancreas stained with hematoxylin–eosin from euthyroid (C), hypothyroid (D), euthyroid + hypercaloric diet (E), and hypothyroid + hypercaloric diet (F) 40×. The horizontal line represents 50 µm. The presence of pancreatic acini (PA) and islets of Langerhans (IL) are observed. In addition, the quantification of the number of islets (G) and cellularity in the islets (H) is presented. Data represent the mean ± s.e. (*n*  = 7); a ≠ b ≠ c ≠ d *P<* 0.05, equal letters indicate that there is no difference between groups, and different letters indicate the statistical difference. RM two-ANOVA and Student–Newman–Keuls *post hoc* to insulin resistance, glucose tolerance, number of islets, and cellularity in islets. The AUC by Mann–Whitney *U*-test.

The functional test of the endocrine pancreas is shown in Fig. 2, hypothyroid animals fed a chow diet presented insulin resistances without changes in the glucose tolerance and the endocrine pancreas morphometrical study. However, animals fed a hypercaloric diet had insulin resistance, glucose tolerance, reduction in the number of Langerhans islets, and an increase of its cellularity; moreover, the most affected was the congenital hypothyroid group.

**Figure 3:**
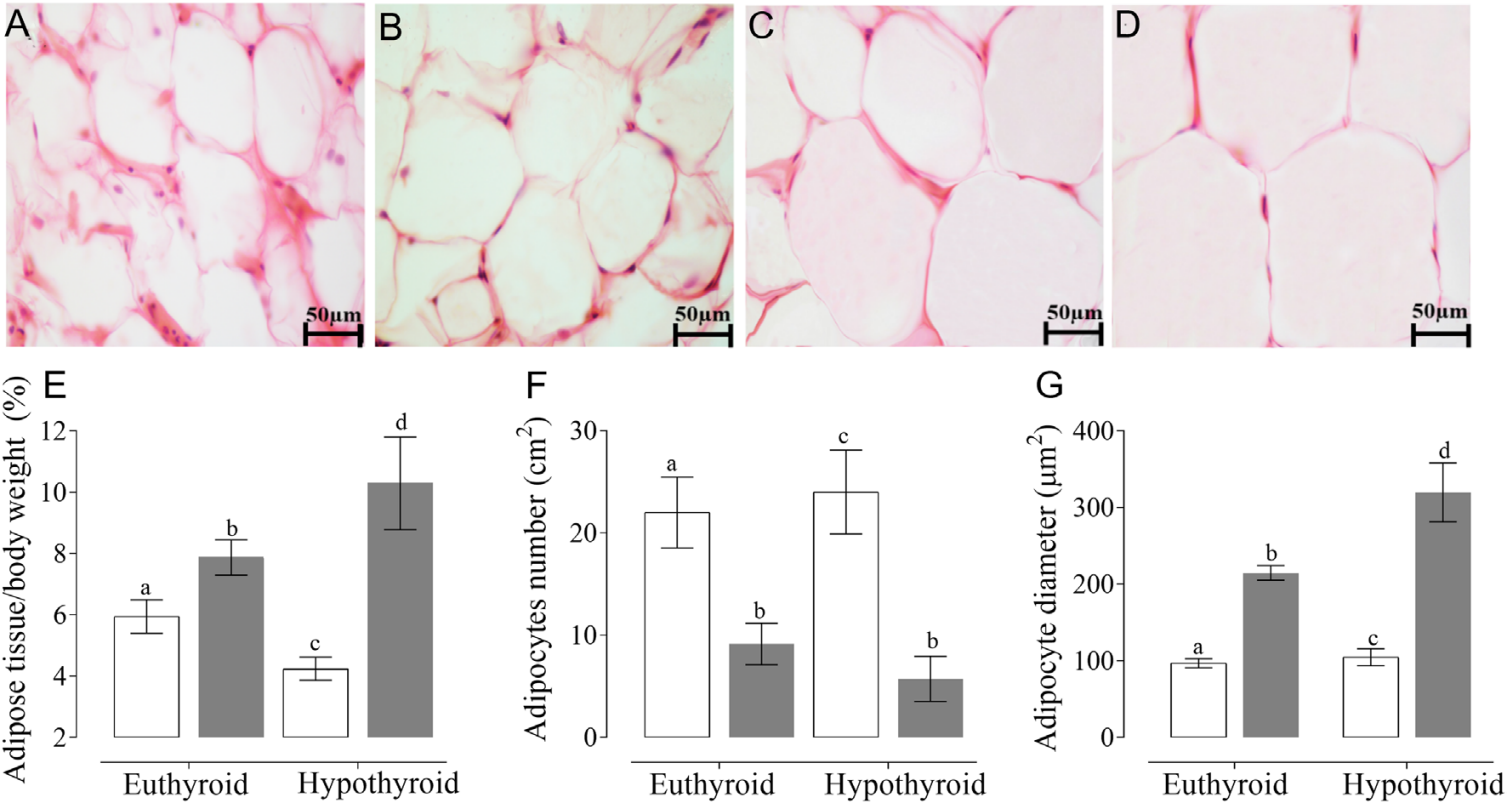
Adipose tissue photomicrography from euthyroid (A), hypothyroid (B), euthyroid + hypercaloric diet (C), and hypothyroid + hypercaloric diet (D) rats 40×, stained with hematoxylin–eosin. The horizontal line represents 50 µm. The rounded shape of the adipocytes can be observed with the presence of a peripheral nucleus. The graphs represent the adipose tissue/body weight percentage (E), adipocyte numbers (F), and the adipocyte diameter (G); a ≠ b ≠ c ≠ d *P<* 0.05*,* equal letters indicate that there is no difference between groups, and different letters indicate statistical difference. RM two-ANOVA and Student–Newman–Keuls *post hoc.*

The adiposity levels and the morphological analysis of the adipose tissue are shown in Fig. 3. Congenital hypothyroidism generated lower adiposity levels, and the adipose tissue presented a greater number of adipocytes per cm^2^ and greater diameter compared to the euthyroid control group. When administering a hypercaloric diet, the adiposity levels increased and the morphological analysis showed a reduction in the number of adipocytes per cm^2^ with a considerable increase in the cell diameter, the changes were exacerbated in hypothyroid animals.

## Discussion

Thyroid hormone (T_3_) is one of the responsible hormones for bodyweight regulation because it modulates long-term bodyweight programs related to the leptin and insulin pathways (15, 24, 25). Rats fed a hypercaloric diet for 40 weeks had the highest hyperleptinemia levels with physiological leptin resistance. But usually, the leptin resistance can only be demonstrated by a molecular evaluation of hypothalamic SOCS3 overexpression because energy intake does not change (14, 15). The results obtained show that a mild disturbance in the lipid contents of the diet produces metabolic damage, causing dyslipidemia, hyperleptinemia, and alterations in cardiovascular risk markers as Castelli index I and II. All of them indicated a cardiovascular and atherogenic risk increase.

The hypercaloric diet alters glycemic regulation, causing hyperglycemia, hyperinsulinemia, high levels of HOMA-IR index, insulin resistance, and glucose tolerance. Also, the histological analysis of the pancreas revealed cytomorphological alterations related to the diabetic state (a decrease in the number of Langerhans islets with an increase in its cellularity). Hypercaloric diet caused hyperglycemia and hyperleptinemia that stimulated β-cell proliferation (26), this compensatory mechanism underwent structural modifications in β-cell in response to the circulating hyperglycemia, increasing insulin secretion. This feedback loop caused cellular hyperplasia (27). When hyperglycemia, hyperinsulinemia, and circulating free-fatty acids persist for a long period, it enhances the oxidative stress process and cell death in the pancreatic β-cells. If the pancreatic tissue lost its physiological function, it generates glucose intolerance, insulin resistance, and finally, the pancreatic dysfunction that causes T2D (28, 29).

In addition, pancreas functioning is modulated by T_3_ action (30). During the intrauterine stage, it modulates pancreas growth and maturation (31) and inhibits β-cell proliferation in a dose-dependent manner (26). Thus, when congenital hypothyroidism is presented, it alters the intrauterine pancreas programming having long-term consequences when animals are fed with a hypercaloric diet (24).

Adipose tissue is one of the main targets for thyroid hormone action, playing a central role in bodyweight, glucose regulation, and the storing of energetic metabolites (32, 33). T_3_ regulates adipogenesis and related processes such as lipogenesis and lipolysis (34). Congenital hypothyroidism compromises the correct adipose tissue programming affecting its functioning under normal physiological conditions (35, 36).

As expected, the adiposity percentage increased in animals fed a hypercaloric diet, changing the size of the adipocytes. Although, the total adipocytes presented in the adipose tissue are determined in the early life stages, and they remain constant throughout the development. In experimental models, it has been shown that adult rats do not lose adipocytes when they are starving or acquire new adipocytes in a period of rapid gain in bodyweight (37). The increase in the size of adipocytes rises adipokines secretion such as leptin and adiponectin modifying the long-term bodyweight regulation pathways as ObRb-STAT3 leptin signaling (38). In addition to higher expression and secretion of inflammatory cytokines causing a chronic inflammation state that constitutes an important mechanism for the development of insulin resistance, dyslipidemia, and cardiovascular complications observed in the context of obesity (39).

This could explain the increase in the incidence of T2D in developing countries like Mexico with a high poverty and marginalization rate, in which the metabolism of the people is adapted to ‘thrifty genotype’ (2) and there is a willingness to consume foods with a high energy value. Also, nowadays in Mexico, the National Health System only screens for congenital hypothyroidism in neonates, but we believe that it is crucial to have a thyroid state screening before or during pregnancy to avoid erroneous metabolic and thyroid gland programming and the development of cardiometabolic diseases in adulthood.

The results presented allow us to conclude that the alterations produced by congenital hypothyroidism led to permanent alterations in the metabolic programming causing dysfunctions in the functioning of the pancreas and adipose tissue, and when the congenital hypothyroid animals are fed a hypercaloric diet, they develop T2D earlier and with worsening prognosis than euthyroid animals, having a lower survival rate under the same conditions.

## Declaration of interest

The authors declare that there is no conflict of interest that could be perceived as prejudicing the impartiality of the research reported.

## Funding

This study was partially supported by SIP-IPN (20200521, 20201091, and 20200493). We thank Instituto Politécnico Nacional. The researchers are fellows of EDI, COFAA, and SNI.

## Statement of ethics

Experimental procedures described in this study follow the Mexican Official Standard NOM-082-ZOO-1999 as well as the Guide for the Care and Use of Laboratory Animals from the National Research Council (US) Committee. Also, the protocol received approval from the Internal Bioethics Committee (CEI-ENCB) with the number approbation CEI-ENCB 030/2019.

## Author contribution statement

J A Tapia-Martinez conceptualized and designed the study, and wrote the manuscript. M Franco-Colin performed the statistical analysis. V Blas-Valdivia carried out the histological study. E Cano-Europa analyzed and interpreted data, and carefully revised the manuscript. All the authors have read and approved the manuscript.
